# Towards a Communication Brain Computer Interface Based on Semantic Relations

**DOI:** 10.1371/journal.pone.0087511

**Published:** 2014-02-07

**Authors:** Jeroen Geuze, Jason Farquhar, Peter Desain

**Affiliations:** Radboud University Nijmegen, Donders Institute for Brain, Cognition, and Behaviour, Nijmegen, The Netherlands; University of Maryland, College Park, United States of America

## Abstract

This article investigates a possible Brain Computer Interface (BCI) based on semantic relations. The BCI determines which prime word a subject has in mind by presenting probe words using an intelligent algorithm. Subjects indicate when a presented probe word is related to the prime word by a single finger tap. The detection of the neural signal associated with this movement is used by the BCI to decode the prime word. The movement detector combined both the evoked (ERP) and induced (ERD) responses elicited with the movement. Single trial movement detection had an average accuracy of 67%. The decoding of the prime word had an average accuracy of 38% when using 100 probes and 150 possible targets, and 41% after applying a dynamic stopping criterium, reducing the average number of probes to 47. The article shows that the intelligent algorithm used to present the probe words has a significantly higher performance than a random selection of probes. Simulations demonstrate that the BCI also works with larger vocabulary sizes, and the performance scales logarithmically with vocabulary size.

## Introduction

A Brain Computer Interface (BCI) [1] is a system that translates measured brain activity into machine commands without the use of any muscles or peripheral nerves. It could for instance allow someone to control a wheelchair or send commands to a computer. In theory this seems rather straightforward; a subject or patient performs a certain mental task and the computer tries to detect what the subject is doing. In practice, however, the signals are often measured outside the skull using, for instance, Electroencephalography (EEG) [2], [3]. Because of the electrical conductive properties of the dura, skull and scalp, the signal measured is a more indistinct and low dimensional version of the signal actually produced by the brain. Also, the signal produced by the brain is a coded signal, which needs to be decoded by the BCI system. The output of a BCI can be used for multiple purposes. Here, the focus is on communication.

A number of BCIs have already been developed with communication in mind. The best researched of these is the visual speller [4]. There the subject spells characters by looking at them on the screen. The letters are accentuated, often by a change in brightness, and subjects are asked to count the number of accents on the character they want to select. The accentuation of the target character elicits a P300 response [5] in the subject’s brain. This response is exploited by the BCI to decode the intention of the subject. However, visual spellers work best when subjects are still able to foveate the character they want to select, allowing the BCI to also use brain responses in the primary visual cortex. In the last few years, more research has been conducted into transforming the visual speller into a BCI that can also be used by patients that are not able to move or focus their eyes anymore. Treder and Blankertz [6] looked at an alternative visual speller design, where foveation was not necessary. Other researchers have focussed on other modalities besides the visual modality, e.g., an auditory speller [7], [8], a speller where the auditory and visual modality are combined [9], a tactile speller [10] and a speller based on imagined movement [11].

However, all of these communication BCIs are based on spelling out the message to be communicated character by character. This article describes a communication BCI that is based on word selection by utilising semantic relations between words. By presenting many words in rapid succession and collecting responses to those words that are related to the word to be communicated (prime word), the BCI is able to decode this prime word. It builds upon earlier work, which shows that the semantic priming response, i.e., the response that differs when words are related versus unrelated, can be detected at the single trial level [12]. A first attempt at building this BCI utilised this semantic priming response. However, due to differences between the offline study in Geuze, van Gerven, Farquhar, and Desain [12] and the online implementation, the single trial detection was reduced to chance level. These differences are explained in more detail in the discussion section. It was concluded that a more robust brain signal was necessary to operate the BCI. Actual movement was chosen for three reasons. First, actual movement provides a strong brain signal that can be classified with high accuracy. Second, when this BCI would be used by paralysed patients they would attempt movement. Blokland et al. [13] has argued that the neural signal generated by attempted movement more closely resembles the neural signal generated by actual movement in non-paralysed subjects. Last, by having subjects press a button when they see a related word, more information about the actual brain activity is collected, than when only relying on semantic relations predicted from another source. To get an idea about the performance of this BCI using imagined movement, the best performing subject redid the experiment with imagined movement instead of actual movement.

The BCI works by presenting 100 probe words in rapid succession (one every 1.35 seconds). From the 150 possible prime words subjects select a word they want to communicate, and keep this word in mind. They press a button every time they are presented with a probe word that is related to their selected prime word. The probe words that are presented are a subset of the prime words that the BCI is able to detect, where it is possible that the same probe word is presented multiple times. The BCI collects the subjects EEG (electroencephalogram) data and uses a binary classifier to determine whether the brain’s response to the probe word includes a movement response or not. By combining the classification results for each presented probe word with a database containing semantic relations between all prime and probe word combinations, the system attempts to identify the intended prime word.

In the study described here, there are 150 prime words and the same set of possible probe words. Randomly selecting a probe word to be presented next could suffice with such a small number of words. However, when using more possible words, this quickly becomes problematic. To solve this, an algorithm was developed that selects the probe word in an informed way. The algorithm uses the decoding state of the BCI and selects the probe word which, when presented, would elicit the most information in determining which word is the prime word.

The decoding and probe selection algorithms were implemented and the BCI was tested with 11 subjects in order to answer the following questions: (i) *Is it possible to build a BCI based on semantic relations using an intelligent probe selection algorithm?* (ii) *Does applying a dynamic stopping technique contribute to the performance of this BCI?* (iii) *Does this intelligent selection contribute to the performance of the BCI?*, (iv) *Do the results of the BCI scale to large numbers of prime and probe words?* Post-hoc simulations were used to answer the last two questions. The simulations were performed using the real subject single trial classification results. The collection of the data required to answer the first two questions took more than 2 hours per subject. Therefore it was decided to answer the last two questions with simulations.

## Methods

### Ethics Statement

The procedures used in the experiment were according the Declaration of Helsinki, and all subjects gave written informed consent. The procedures were approved by the Ethical Committee of the Faculty of Social Sciences at the Radboud University Nijmegen.

### Subjects

The electroencephalogram (EEG) of 11 right-handed, native Dutch subjects was measured. Their age ranged from 18 to 28 (M = 22.4, SD = 3.2) and 7 of the subjects were female. All subjects had normal or corrected-to-normal vision and were free of medication and neurological abnormalities. All subjects participated voluntarily and gave written informed consent. All subjects but two (S1 and S5) received a reward in the form of money or study points. One participant (S1) also participated in a previous study [12]. One of the subjects (S2) was observed not to pay attention during the experiment and not perform the task and look around for periods of time. This was confirmed by the data, where the mismatch between expected button presses and actual button presses in the training block was more than 2 standard deviations higher than the average over subjects. On these grounds, this subject was not included in the analysis.

### Procedure

The experiment consisted of five blocks. First, a practice block for the subjects to become acquainted with the task. Second, a training block where data are gathered to train the classifiers. Then, two test blocks where the classifiers are applied to the data and feedback is given about which word the subject saw as a prime. Last, there is a post-training block with the same properties as the training block, but shorter. This post-training block is used to determine any time-based deterioration of classifier performance due to non-stationarities in the data.

**Figure 1:**
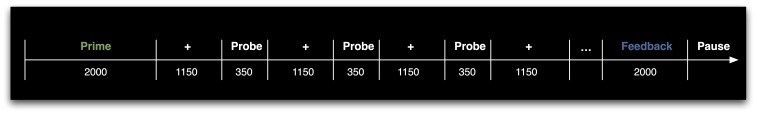
Design. Basic design of the experiment.

Subjects were seated in a comfortable chair in front of a computer screen. First the prime word was presented in a green colored font for 2000 ms. Then, a fixation cross was shown for 1150 ms, followed by a probe word for 350 ms and another fixation cross for 1150 ms, all in a white colored font. The probe and fixation cross were repeated until the total number of probes for the given prime word had been reached. A graphical representation can be seen in Figure 1. Subjects were instructed to press a button with their right index finger when they found that a probe word was related to the prime word they were shown earlier. They were instructed to keep their finger on the button throughout the experiment to minimize movement artefacts. Their EEG was measured during the experiment. The button press itself was not used during the online analysis, which were solely based on the recorded EEG activity.

In the training block 36 prime words are presented each followed by 5 related probes and 10 unrelated probes in random order. After presenting three prime word sequences consecutively, the subject can take a break. In a test block, 6 prime words are presented each with 100 probes. The probe selection is performed by the algorithm explained in detail in the decoding section below. Since the prime word sequence is too long to present at once (100 probes), subjects can take a break after 30 probes. After pressing a button to continue, the prime word is presented again to remind the subject. When all 100 probes have been presented feedback is given about which word the decoding algorithm selected based on the subject’s brain activity. The feedback is given in a blue colored font. The post-training block is similar to the training block, only with 12 prime words instead of 36.

### Stimuli

Stimuli consisted of words drawn from the Leuven association dataset [14]. This dataset was constructed by having subjects perform a continuous word association task. The cues were constructed by the researchers, while the associated words were generated by the subjects. For each word pair their association strength was determined by dividing the number of times the response was given to that particular cue by the total number of responses to that cue.

For the training stimuli 36 prime words were selected. For each of these prime words, 15 probe words were matched, 5 which were related and 10 which were unrelated. For the related probe words, only words with a high association strength were chosen (>.14). For the unrelated words, words were selected with an association strength of 0. This resulted in 180 related probe words (M  = 0.24, SD  = 0.073) and 360 unrelated probe words (M  = 0, SD  = 0).

**Figure 2:**
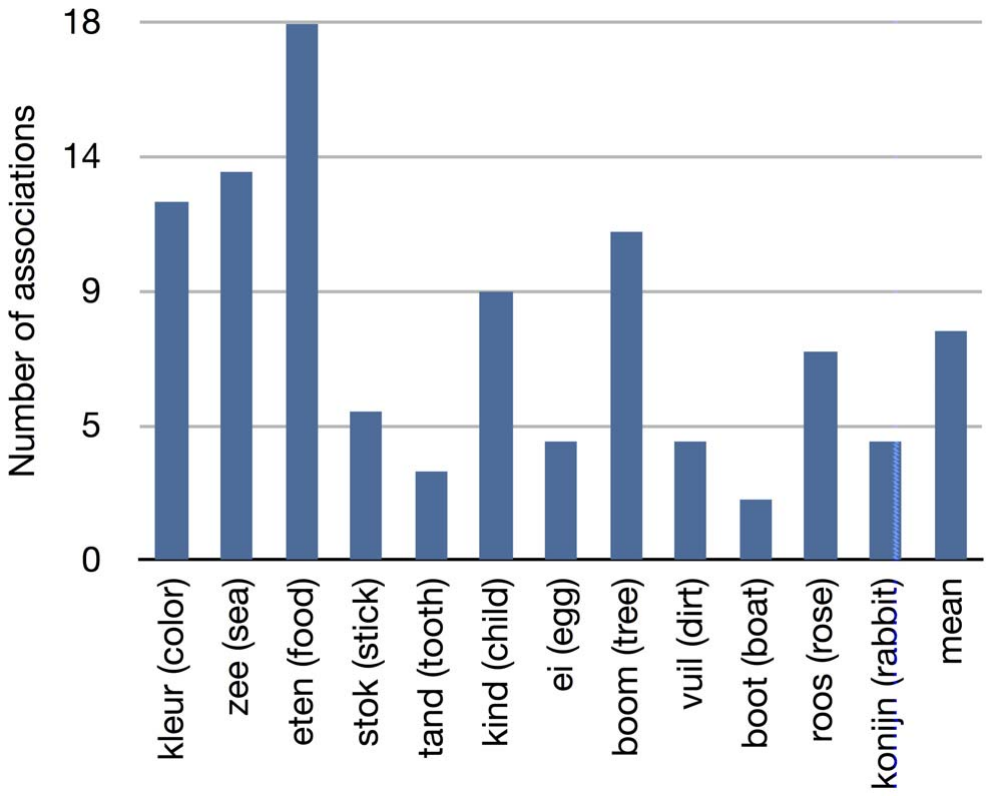
Number of associations. Number of associations for prime words in the test blocks.

**Figure 3:**
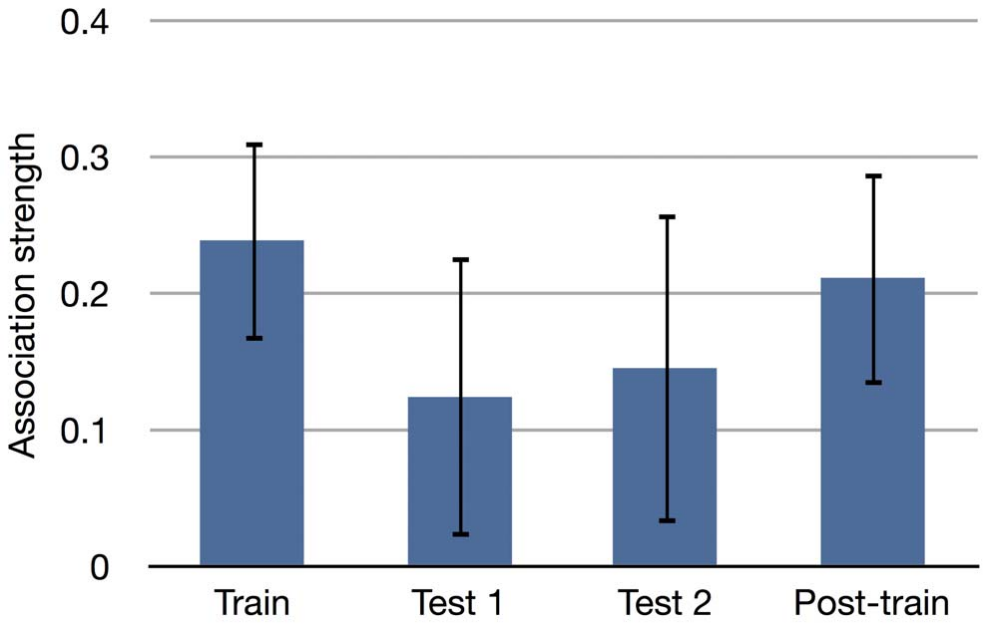
Association strength. Average association strength per block. The error bars indicate the standard deviation.

For the test stimuli a subset of the Leuven association dataset was constructed by selecting the 150 words with the most connections, i.e., number of related words. From this subset, 12 words were selected to be presented as primes. Three primes with a high number of connections (color, food, sea), three primes with a low number of connections (stick, tooth, child), and six random prime words (egg, tree, filth, boat, rose, rabbit). For the exact number of connections per prime word, see Figure 2. Seven of these words were seen as prime before, 6 in the training block (color, food, child, egg, tree, boat) and 1 in the practice block (sea). One of the prime words also occurred in the post-training block (tooth). For the probe words a selection from the constructed subset was used, for more information on probe selection see the decoding section below. The prime word could also occur as a probe word. Because this did not occur in the Leuven dataset, the association strength of a prime with itself was set to the maximum association value in the dataset. The average association strength of the probe words can be seen in Figure 3.

The post-training stimuli were constructed in the same way as the training stimuli, but for only 12 prime words. This resulted in 60 related probe words (M  = 0.21, SD  = 0.078) and 120 unrelated probe words (M  = 0, SD  = 0).

An overview of all the stimuli can be found in the supporting information: File S1 (Stimuli).

### Equipment

The stimuli were presented with Psychtoolbox [15]–[17] version 3.0.8 running in Matlab 7.4. The stimuli were displayed on a 17′′ TFT screen, with a refresh rate of 60 Hz. The data were recorded using 64 sintered Ag/AgCl active electrodes using a Biosemi ActiveTwo AD-box and sampled at 2048 Hz. The electrodes were placed according to the 10/20 electrode system [18]. The EEG was recorded in an electrically shielded room. The EEG offset for each channel was kept below 25 *μ*V. A button box was used to allow participants to start the next sequence and indicate whether a probe word was related. Brainstream (http://www.brainstream.nu/), a toolbox for running online BCI experiments was used to coordinate the presentation of the stimuli, managing the EEG data and running the online classification analysis pipelines.

### Data Availability

The data is stored locally, in multiple locations, which are regularly backed-up. The anonymous data is available in on request from the lead author.

### Data Analysis

The analyses were performed by Brainstream (http://www.brainstream.nu/), the plotting of the grand average results was performed using the Fieldtrip toolbox [19].

A part of the analysis pipeline for both Event Related Potentials (ERP) and Time Frequency Representations (TFR) was common, therefore, these steps were performed on the continuous EEG data before they were sliced from 0–1350 ms after probe onset. This common pipeline first temporally down-samples to 256 Hz and removes linear trends. Bad channels were detected and removed and eye artifacts were removed by de-correlating the EEG and EOG channels. To maintain a consistent channel set the removed channels were reconstructed using spherical spline interpolation [20]. These data were then sliced and used as input for the two classification pipelines.

To classify the data based on the evoked single trial ERP, the training data were sliced from 0–1350 ms after probe onset. Outlying trials, i.e., a trial where the power differed by more than 3 standard deviations from the trial median, were removed. A common average rereference was calculated and the data were filtered between 0.1 Hz and 10 Hz. This was then used to train a binary L2 regularised logistic regression classifier. The related and unrelated classes were balanced by selecting a random subset from the unrelated class to match the number of trials in the related class. This was done to prevent the classifier from always selecting the dominant class. The above mentioned steps (except the outlier removal) were also performed per single trial in the online test blocks, after which the trained classifier was applied to the data, resulting in a decision value. The same preprocessing steps (except the balancing) were performed to obtain the data in the grand average ERPs. To test for significant differences between brain responses to related probes and to unrelated probes the cluster-based non-parametric statistic described by Maris and Oostenveld [21] was used. This test corrects for the multiple comparisons problem by incorporating a permutation test.

To classify the data based on the induced response a single trial time frequency representation was used. The training data were sliced by brainstream from 0–1350 ms after probe onset. Outlying trials were removed, after which a rereference based on surface laplacian was applied to increase spatial specificity. The time frequency representation was calculated with a hanning window of 500 ms with an overlap of 50%. Then the frequencies of interest were selected (8–24 Hz) and the resulting data were used to train another binary L2 regularized logistic regression classifier. The related and unrelated classes were balanced by selecting a random subset from the unrelated class to match the number of trials in the related class. The above mentioned steps, excepting the outlier removal, were performed on the online single trials in the test blocks, before applying the trained classifier. As before, the data that were used to train the classifier was also used to plot the grand average TFRs. To test for significant differences between the conditions the cluster-based non-parametric statistic was used with the same settings as for the ERP analysis.

A combined classifier was obtained by adding the decision values of the individual classifiers. The classifiers are calibrated to produce valid estimates of the likelihood of a button press given the features. Thus, adding decision values in this was is equivalent to a bayesian information combination under the assumption of conditional independence of the classifiers.

### Decoding

In the decoding algorithm, classifications of multiple probes are combined to determine the prime word the subject is trying to communicate. If the codebook **C** is a matrix of *n* primes by *m* probes, indicating for each prime-probe-combination whether they are related or not. At the end of the sequence the prime word with the highest probability is selected by
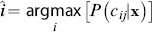
(1)where **x** is a vector of decision values, one for each probe. The probability for each target is calculated by combining the codebook and the individual decision values for each presented probe word:
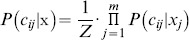
(2)where *Z* is an irrelevant normalisation constant, and where the probability a probe belongs to the class indicated in the codebook, given the decision value of the classifier is given by the logistic function,
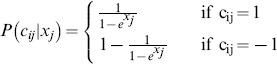
(3)where related is assigned as the positive class, indicated by 1, and unrelated is assigned as the negative class, indicated by −1.

The probe to be presented next in the experiment is the probe for which the probability that the subject recognises it as related is closest to .5:
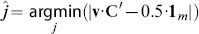
(4)where the codebook **C** again indicates which prime-probe-combinations are related and where **v** is the vector with the probabilities for each prime word based on the probes that have been presented so far:



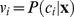
(5)Choosing the probability for a probe close to .5 optimizes the amount of information transmitted by the response:
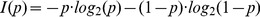
(6)


### Post-hoc Analysis

A number of post-hoc analysis were performed to compliment the data obtained during the experiment. First an early stopping method was applied to determine at which point time the prime word sequence could be stopped without losing accuracy. A number of methods are discussed in Schreuder et al. [22]. Three of these methods (fixed number, Jin et al. [23], and Höhne et al. [8]) and an additional method not mentioned by Schreuder et al. [22] were compared with not stopping. The additional method, thresholding the probability of a target given the data, as given in Equation (2), at 0.95, performed best and was selected for determining the stopping point. The last method The early stopping was first applied to the data gathered from the experiment, and later to all subsequent post-hoc analyses.

To obtain data that are too time-consuming to gather from subjects, post-hoc simulations were performed. The algorithm detailed in the decoding section above was implemented, where the classifier decisions were drawn from the decision values that were gathered during the experiment. Simulation results are obtained by simulating each word 100 times (iterations) for each subject and averaging over iterations, items and subjects, i.e., each number is the mean of 12.000 simulated prime sequences. The decision values were pooled per subject per block into a related and unrelated pool, based on the codebook constructed from the association database, i.e., not using the button presses.

The results from the experiments were simulated, by using the same parameters, to compare the simulation results to the data obtained in the experiment. However, where the experiment yielded one value per subject, per word, the simulations yielded 100.

To determine whether the information-based probe selection performs better than random probe selection, a simulation was run where the probes were selected at random.

To investigate whether the algorithm scales to larger numbers of prime words, the simulation was run with 150, 500, 1.000, 2.500, and 10.000 prime words. (For the 10.000 prime words condition in fact only 9.270 prime words were used because that is the size of the Leuven dataset. For communication convenience we use 10.000 or 10k.) In the experiment, 150 words were used as both prime words and probe words, resulting in a codebook (**C**) of 150×150. As a baseline for the scale to larger number of prime words a simulation was run where the maximum number of probe words were used (10.000), i.e., in the comparison only the number of prime words changes. The 150 prime words used in the experiment were always included and a random set of probe words was selected to supplement the total number of prime words to the required amount.

To evaluate the results of the post-hoc analyses, a number of measures were used: rank, proportion correct, number of probes, and Information Transfer Rate (ITR). The rank is defined as the position in the list of targets when sorted on their probability (see Equation (2)). The proportion correct can be indicated in three ways. The actual proportion correct (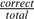), the proportion related correct, where words that are related to the prime word are also counted in the numerator, and proportion in rank top 10, where words that have rank 1–10 are also counted in the numerator. The number of probes is simply the amount of probes that are used before reaching the stopping criterium. The Information Transfer Rate (ITR) is a measure that is often used to compare algorithms, because it incorporates accuracy, number of classes, and the time per classification. Wolpaw, Ramoser, McFarland, and Pfurtscheller [24] defined the ITR for a Brain Computer Interface as:
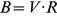
(7)Where *B* is the ITR in bits per second, *V* is the amount of classifications per second, and *R* is defined as:
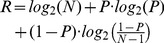
(8)ITR is often reported in bits per minute by multiplying *B* with 60.

## Results

### Grand Average Results

**Figure 4:**
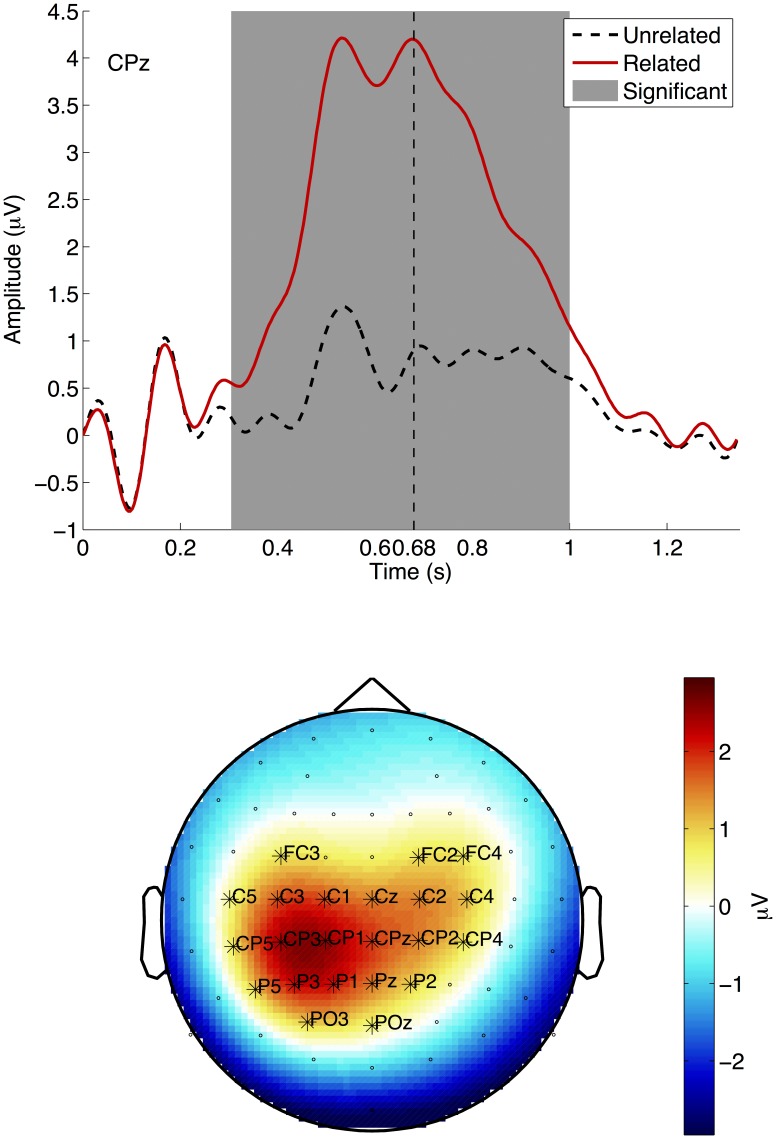
Grand Average ERP. Grand average Event Related Potential (ERP) results for the training block. Top: the ERPs for the related (solid red) and unrelated (dashed black) conditions. The grey area indicates where the conditions differ significantly. The dashed vertical line indicates the average reaction time, i.e., when the subjects pressed the button. Bottom: The distribution over the scalp of the significant difference (related – unrelated) averaged over the grey area of the top panel (260ms –1000 ms). Asterisks indicate for which channels the effect is significant.

The grand average ERP results can be seen in Figure 4. The figure shows the ERPs for the related condition (in solid red) and unrelated condition (in dashed black) for channel CPz for each of the training block. The grey area indicates where the two conditions differ significantly, as indicated by the cluster-based non-parametric statistic described by Maris and Oostenveld [21]. The vertical dashed line indicates the grand-average reaction time, i.e., when subjects pressed the button. Channel CPz was chosen as a representative channel. The topo-plots of the time window indicated by the grey area in the ERP plot show the distribution of the effect over the scalp. Channels indicated with an asterisk are significant in this time window.

**Figure 5:**
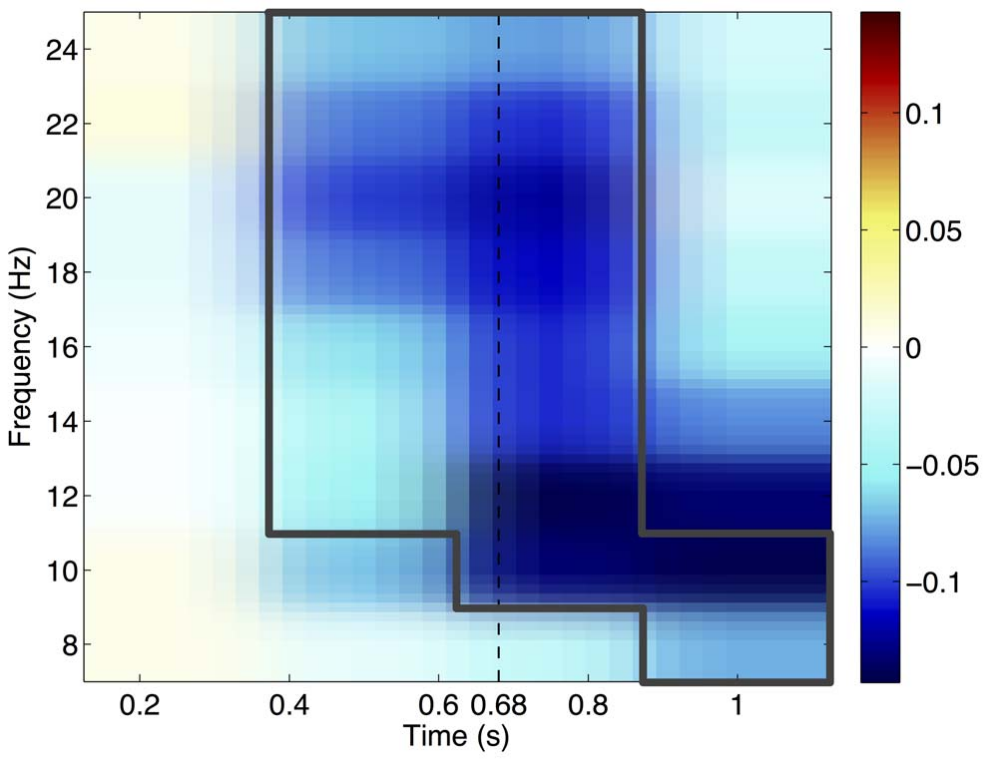
Grand Average TFR. Grand average TFR results for the training block for channel C3. The data shown here is a normalised difference between the related and unrelated conditions, obtained by 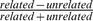. The grey box indicates in which parts of the figure the difference between the two conditions is significant. The vertical dashed line indicates the grand average reaction time, i.e., when the subjects pressed the button.

The grand average Time Frequency Representation (TFR) results are shown in Figure 5. Channel C3 was selected as a representative channel because right-hand motion is most strongly visible above the motor-cortex in the contra-lateral hemisphere. The data in Figure 5 are a normalised difference between the two conditions, obtained by first subtracting the TFR data from the unrelated condition from the related condition and then dividing the result by the sum of the two conditions (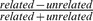). The area within the grey box is where the two conditions are significantly different, as indicated by the cluster-based non-parametric statistic described by Maris and Oostenveld [21].

### Classification Results

An overview of the single trial classification results is shown in Table 1. All reported accuracies are significantly different from chance level (0.5), with p-value of <.001, based on a binomial test [25], Ch. 17. These classification results are based on the labels that are taken from the Leuven dataset.

**Table 1:** Classification accuracies.

	Train	Test 1	Test 2	Post-train
S1	85%	75%	73%	80%
S3	75%	66%	65%	80%
S4	87%	74%	77%	87%
S5	74%	65%	65%	76%
S6	79%	62%	64%	70%
S7	75%	60%	59%	67%
S8	74%	61%	64%	70%
S9	73%	65%	63%	66%
S10	73%	66%	69%	83%
S11	88%	62%	66%	83%
Mean	78%	66%	67%	76%
IM	76%	66%	67%	72%

Single trial classification accuracies, based on relatedness labels from the Leuven dataset. All classification accuracies differ significantly from chance level (0.5) with a p-value of <.001.

**Figure 6:**
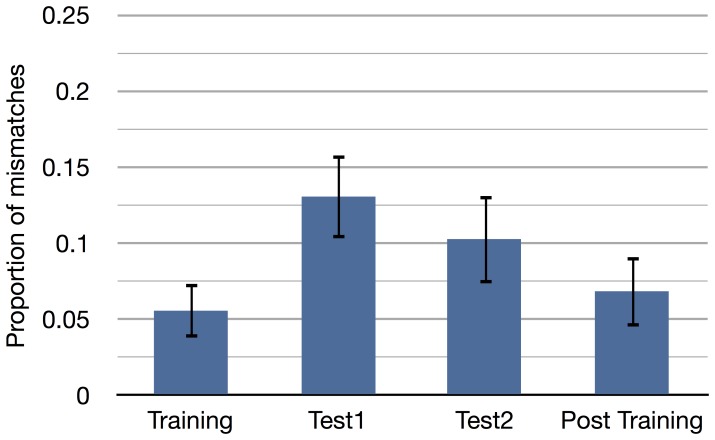
Mismatch. Mismatch between codebook based on Leuven association dataset and button presses. Only the unique mismatches were counted. Error bars are corrected for a within subject design [31], p. 361–366.

To investigate how well the Leuven dataset represents the associations by the subjects and whether that is influenced by the difference in association strength per block (shown in Figure 3), the mismatch between the labels as given by the Leuven dataset (used during the experiment) and the labels that were derived from the button presses of the subjects during the experiment was calculated. The average proportion of mismatched labels per block can be seen in Figure 6. Because in the test blocks, some prime-probe combinations may occur multiple times, only the mismatch for unique combinations it calculated.

An overview of the decoding results can be found in Table 2. It shows the proportion correct in the situation where all 100 probes are used (Full) and in the situation where early stopping is applied (Stop). Asterisks indicate whether the accuracy is significantly different from chance level (1/150, 0.00667), based on a binomial test.

**Table 2:** Decoding results.

	Full	Stop	Probes
S1	83% **	42% **	29
S3	50% **	58% **	36
S4	50% **	42% **	28
S5	25% *	33% **	58
S6	8%	25% *	54
S7	8%	17%	52
S8	42% **	58% **	66
S9	33% **	33% **	75
S10	42% **	50% **	43
S11	42% **	50% **	33
Mean	38% **	41% **	47
IM	58% **	33% **	34

First two columns indicate proportion correct, last column indicates the number of probes used to obtain the accuracy for the *stop* condition, for the *full condition* this is always 100. Asterisks indicate whether the proportion correct differs significantly from chance level (1/150, 0.00667). * indicates .001 < p <.05, ** indicates p <.001.

### Post-hoc Simulation Results

**Figure 7:**
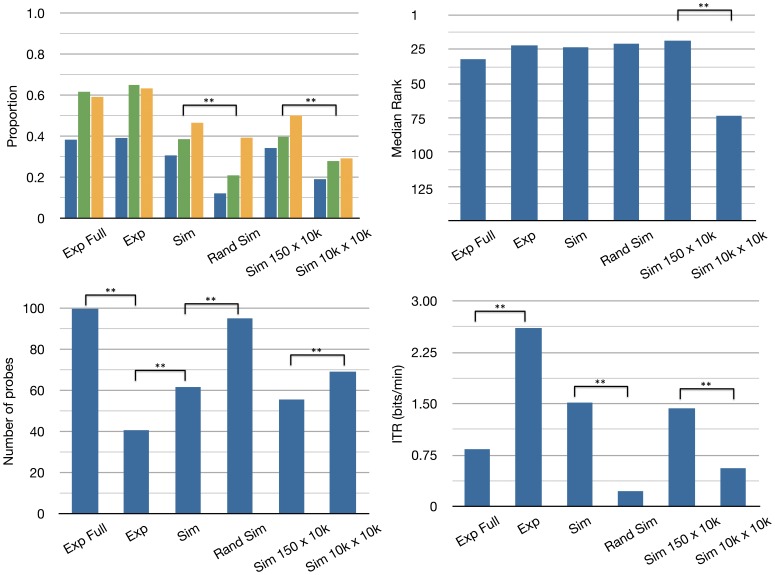
Post-hoc simulations. Results for post-hoc simulations: Exp Full: the experiment using the full number of probes, Exp: results from the experiment with early stopping, Sim: simulation results with early stopping, Rand Sim: simulation with random probe selection and early stopping, Sim 150×10k: simulation with 150 targets and 10.000 probes with early stopping, Sim 10k×10k: simulation with 10.000 targets and 10.000 probes with early stopping. Top-left: Proportion correct, related correct and in top 10. Top-right: Rank, the rank for the last analysis (Sim 10k×10k) is scaled by dividing by 61.8. Bottom-left: Number of probes. Bottom-right: Information Transfer Rate. * indicates .001 < p <.05, ** indicates p <.001.

The results for the post-hoc simulations can be found in Figure 7. It shows the performance on the four measures mentioned earlier: proportion correct (top-left panel), rank (top-right panel), number of probes (bottom-left panel), and Information Transfer Rate (ITR) (bottom-right panel). The different simulations are arranged on the x-axis. From left to right: (i) the results from the experiment using the full number of probes (Exp Full), (ii) the results from the experiment with early stopping (Exp), (iii) simulation results with early stopping (Sim), (iv) simulation with random probe selection and early stopping (Rand Sim), (v) simulation with 150 targets and 10.000 probes with early stopping (Sim 150×10k), (vi) simulation with 10.000 targets and 10.000 probes with early stopping (Sim 10k×10k).

To determine whether the simulation results differ significantly, four Bonferroni corrected one-way repeated measures ANOVA’s were performed, with factor condition with the six analyses as levels. When the ANOVA was significant, bonferroni corrected post-hoc contrasts were performed using Student’s dependent samples t-test. The contrasts of interest were: Exp Full vs Exp, Exp vs Sim, Sim vs Rand Sim, and Sim 150×10k vs Sim 10k×10k. Only the significant contrasts are reported below.

There was a significant difference in proportion correct between the six analyses, F(5,45)  = 13.8, p <.001, 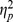  = 0.353. The post-hoc contrasts showed that the proportion correct with intelligent probe selection (M  = 0.307, SD  = 0.14) is significantly higher than the proportion correct with random probe selection (M  = 0.122, SD  = 0.0875), *p*(9)  = 6.79, p <.001. It also showed that the proportion correct in the simulation with 150 targets and 10.000 probes (M  = 0.344, SD  = 0.142) is significantly higher than the proportion correct in the simulation with 10.000 targets and 10.000 probes (M  = 0.191, SD  = 0.108), *t*(9)  = 6.61, p<.001.

There was a significant effect on rank for the six analyses, F(5,45)  = 24, p<.001, 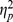  = 0.568. Post-hoc contrasts showed that the rank in the simulation with 150 targets and 10.000 probes (M  = 18.6, SD  = 18.4) is significantly higher than the rank in the simulation with 10.000 targets and 10.000 probes (M  = 73.4, SD  = 24.7), *t*(9) = −9, p<.001.

There was also a significant difference in the number of probes used in the different analyses, F(5,45)  = 52.9, p<.001, 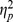  = 0.635. The post-hoc contrasts showed that the number of probes used in the experiment without early stopping (M  = 100, SD  = 0) is significantly higher than when applying the early stopping algorithm (M  = 40.7, SD  = 20.1), *t*(9)  = 9.31, p<.001. It also showed that the number of probes used in the experiment with early stopping (M  = 40.7, SD  = 20.1) is significantly lower than the number used in the simulation with early stopping (M  = 61.6, SD  = 20.8), *t*(9) = −7.45, p<.001. Furthermore, the number of probes used with intelligent probe selection (M  = 61.6, SD  = 20.8) is significantly lower than the the number of probes used with random probe selection (M  = 95.1, SD  = 6.78), *p*(9) = −6.72, p<.001. Finally, the number of probes used in the simulation with 150 targets and 10.000 probes (M  = 55.6, SD  = 19) is significantly lower than the number of probes used in the simulation with 10.000 targets and 10.000 probes (M  = 69.1, SD  = 21.4), *t*(9) = −9.77, p<.001.

A significant difference in Information Transfer Rate (ITR, see Equation (5)) was also found, F(5,45)  = 15.2, p<.001, 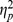  = 0.349. Post-hoc contrasts showed that the ITR in the experiment without early stopping (M  = 0.835, SD  = 0.633) is significantly lower than when applying the early stopping algorithm (M  = 2.6, SD  = 1.59), *t*(9) = −4.91, p  = 0.003. Furthermore, the ITR with intelligent probe selection (M  = 1.52, SD  = 1.52) is significantly higher than the the ITR with random probe selection (M  = 0.222, SD  = 0.255), *p*(9)  = 3.21, p  = 0.042. It also showed that the ITR in the simulation with 150 targets and 10.000 probes (M  = 1.53, SD  = 1.2) is significantly higher than the ITR in the simulation with 10.000 targets and 10.000 probes (M  = 0.668, SD  = 0.758), *t*(9) = −9.77, p<.001.

**Figure 8:**
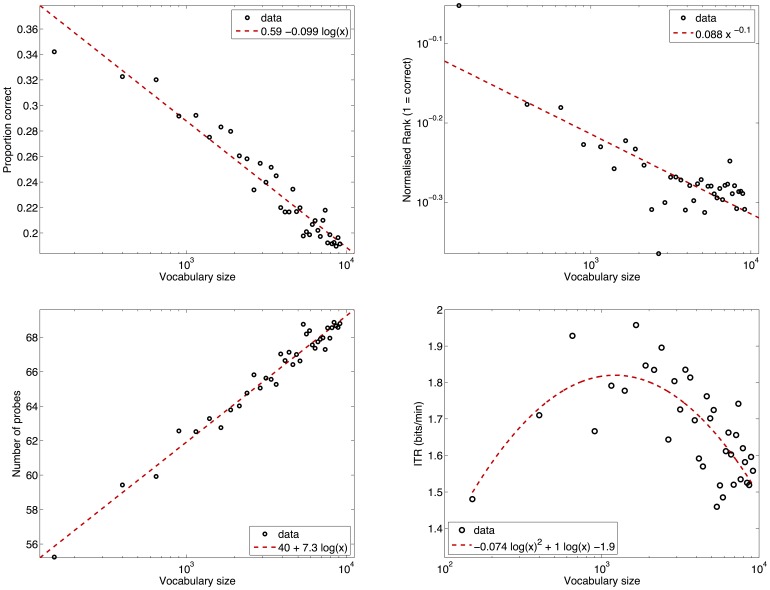
Performace scaling. Scaling of the performance of the BCI with larger vocabularies. The vocabulary size is plotted on the x-axis. The simulation results are indicated by the black circles. A fit of the data is displayed with a dashed red line. Top-left: results for proportion correct, the data were fitted with a logarithmic function. Top-right-panel: simulation rank, the data were fitted with a power law function. Bottom-left: number of probes, data were fitted with a logarithmic function. Bottom-right: Information Transfer Rate (ITR), the data were fitted with a second order polynomial after a log transformation, peaking at a vocabulary size of 1214.

For the scaling to larger vocabularies (more prime words), further simulations were performed, where the number of prime words were gradually increased from 150 to 10.000. The results and a fit of this data can be seen in Figure 8. It shows that the proportion correct decreases logarithmically with vocabulary size with formula 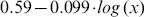, where *x* is the vocabulary size in number of possible prime words. The rank decreases according to a power law function: 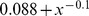. The number of probes until the stopping criterium is reached increases logarithmically approximately according to 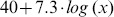. The ITR can roughly be fit with a polynomial after a log(x) transformation: 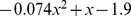, peaking at a vocabulary size of 1214.

## Discussion

The grand average Event Related Potential (ERP) results show a significant P300 effect. The timing of the peak and distribution over the scalp are similar to paradigms eliciting a P300 response [5]. The peak of the response, on average, occurs shortly before the button press, indicating the brain response comes before the button press. The grand average Time Frequency Representation (TFR) results show a significant negative difference in the mu-band, corresponding to the brain activity normally elicited by a finger movement [26]. When looking at the evolution of the difference topography of the ERS in the 10–14 Hz frequency band, it also shows an expected pattern: no difference in the first window (0–250 ms), and then an increasing (negative) difference over motor cortex. The corresponding figure can be found in the supporting information: Figure S2.1 in File S2 (Results). The single trial accuracy on the test items is on average 67%. However, there is a difference of about 10% between the training/post-training and the test items.

The mean decoding accuracy is 38% using the full 100 probes, and 41% when applying early stopping. The Information Transfer Rate (ITR) is 0.835 bits/min with the full 100 probes and 2.6 bits/min with early stopping. The post-hoc simulations show that the performance of the algorithm is significantly better with the intelligent probe selection algorithm than with random probe selection. The simulations also show that the performance scales logarithmically with vocabulary size (number of possible prime words).

A consistent difference in single trial accuracy between training block and test blocks was found. On average, the accuracy of the test blocks was 10% lower than that in the training block. There is often a lower accuracy in test blocks than in the training blocks caused by non-stationarities in the data. The more time between the training block and the test block, the lower the test accuracy. However, this does not seem to be the case here. The accuracy on the post-training block, which occurs furthest away in time from the training block, has a similar accuracy to the training block. There are some differences in the stimuli that might explain the lower accuracy. The mean association strength in the test-items is lower than the strength of the training-items (see Figure 3). Another hypothesis could be that there is a higher mismatch between codebook and button press found in the test sets. This in turn would decrease the single trial accuracy because the labels do not match with when the subject actually moves. However, this does not seem to be the case as the single trial accuracy where the button presses are used as labels, the 10% drop in accuracy remains. Future research efforts should give more insight into the cause of this performance mismatch.

As mentioned before, there is a mismatch between the associations as indicated by the Leuven dataset and the subjective associations of the subjects as indicated by their button presses. This mismatch is shown in Figure 6. The inverse of the mismatch could be seen as a measure of fit of the Leuven dataset. In that case, the overall fit of the dataset is 91%. There is a difference in fit between the training blocks (94% fit) and test blocks (88% fit). This difference could be explained by the difference in association strength between the blocks (see Figure 3). The test blocks have a lower association strength compared to the training blocks. It could be expected that with lower association strengths less people would agree that items are indeed related, decreasing the fit on those particular items.

An early stopping algorithm was applied to the data obtained in the experiment. When the probability of any prime word in the decoding algorithm reached the threshold of.95, the decoding was stopped with that prime word as output. On average the proportion correct did not change, however a significant lower number of probes is used to reach this same accuracy. In other words, it takes less time without affecting the performance, which in turn increases the Information Transfer Rate (ITR) of the BCI.

It was shown here that the intelligent probing algorithm contributes significantly to the performance of the BCI. It increases the accuracy, decreases the number of probes required, and increases the ITR of the BCI. It is also expected, that this difference will become even more pronounced with a larger vocabulary (now 150 words).

Offline simulations found that increasing the vocabulary size resulted in a drop in performance, however this was not proportional to the increase. The proportion correct and number of probes change logarithmically with the vocabulary size. The rank decreases according to the power law and the ITR can be fitted with a polynomial after a log transformation. The maximum of this polynomial occurs at a vocabulary size of about 1.200. This means that the BCI conveys the most information with that vocabulary size.

It has been shown here that the BCI works by measuring subject’s actual movement. According to Blokland et al. [13] actual movement is closer in brain signature to attempted movement, i.e., when paralysed subjects try to make an actual movement, than imagined movement. The subject with the best performance returned to do the experiment again with imagined movement. A comparison between this subject’s data in the actual movement session and in the imagined movement sessions was made. It showed that the ERP results were almost identical between the two conditions. In the TFR, the imagined movement had a similar pattern, but a lower amplitude, which is in line with the previous research [27]. The corresponding figures can be found in the supporting information: Figure S2.2 and Figure S2.3 in File S2. The classification results are also almost identical to the grand average movement results and show that this BCI could also work based on imagined movement.

The codebook used by the BCI, based on the Leuven association dataset [14] is sparse and not optimal. Results show a difference between the associations based on the dataset and the associations as judged by the subjects, i.e., the mismatch mentioned earlier. The single trial classification results could be improved by using the labels given by the subjects during the experiment. However, it is not possible to improve the decoding process by using these labels. In order for the decoding to be fair, all possible combinations of prime and probe words need to be manually labelled by the each subject. With a vocabulary size of 150 words, there are already 11.175 combinations, which would take about 4,5 hours to label. Increasing the vocabulary size to the earlier mentioned optimum 1.200 words, increases the combinations to 719.400 (about 280 hours). So for smaller codebooks, some time could be spent in optimising the codebook to further increase the performance of the BCI.

A way to keep the vocabulary size small is to use context to construct the vocabulary. When the BCI is to be used by a patient who wants to communicate about wishes (e.g., *I would like some coffee*) and feelings (e.g., *my leg hurts*), words needed to communicate this could be selected as the vocabulary. By using this context, the total number of words could be kept relatively small, allowing for a similar performance as reported here, and allowing the patient to manually label all possible combinations for improved performance.

The proposed BCI could be useful for two different groups of patients. First, the group of locked-in patients who are not able to communicate anymore. For these patients, this BCI could be an alternative for the existing (visual) spellers. Instead of spelling a word letter by letter, the word or concept is communicated directly using the semantic relations BCI. Further research is needed to determine which method patients prefer. The BCI would also work when pictures or auditory presented words are used instead of visually presented words. This would open up the application for patients that are not able to read, due to illiteracy or other causes. Second, the group of patients with aphasia, especially the patients where the recognition is still intact, but language production is impaired and spelling itself is impossible or very slow. These patients would not need the brain control. For these patients the button-presses themselves can be used, dramatically increasing the performance of the system. Simulations with perfect classification accuracy show perfect decoding accuracy after about 18 probes, and an ITR of around 23 bits per minute.

A different way to detect concepts or words could result from the work of Huth, Nishimoto, Vu, and Gallant [28], Simanova, Hagoort, Oostenveld, and van Gerven [29], or Schoenmakers, Barth, Heskes, and van Gerven [30]. They attempt to decode concepts, words, or images from the brain by looking at activation patterns measured by functional Magnetic Resonance Imaging (fMRI). Currently this still requires presenting stimuli to the subjects and decoding the response to these stimuli. However in future it may be possible to decode this information when the subject has the stimulus in mind.

## Conclusions

This article shows that (i) it is possible to build a BCI based on semantic relations using an intelligent probing algorithm, (ii) Applying a dynamic stopping technique significantly contributes to the performance of such a BCI, (iii), the intelligent selection algorithm contributes significantly to the performance of the BCI, and (iv) the number of required probes increases slowly (logarithmically) with increasing numbers of probe words and primes.

## Supporting Information

File S1 **Stimuli.** Full list of stimuli. List of all stimuli used in the experiment, including semantic relations.
(PDF)

File S2 **Results.** Additional results. Three figures with additional results.
(PDF)
