# Supplementary material for: Towards a Communication Brain Computer Interface Based on Semantic Relations
Source: PLoS One. 2014 Feb 7;9(2):e87511. doi: 10.1371/journal.pone.0087511 (PMC3917851; doi:10.1371/journal.pone.0087511)
Supplement: File S1 — Stimuli. Full list of stimuli. List of all stimuli used in the experiment, including semantic relations. (PDF) [file pone.0087511.s001.pdf]

S1.1 Training stimuli

Prime	Related probes	Unrelated probes
auto	krik, stuur, garage, rijbewijs, gordel	trouw, rugvin, postpakket, framboos, getuigenis, flatgebouw, breigoed, balletschoen, geluksdag, kruisbeeld
baby	luier, kinderbed, kinderwagen, kinderstoel, ooievaar	praatje, ziel, kobbe, dansvloer, rijweg, reggae, stopteken, belg, hagel, vervuiling
bakker	pistolet, croissant, brood, roomsoes, appelflap	presentatie, schoensmeer, kelp, borduursel, reflex, bles, vacature, ribbe, trom, kookplaat
bal	rugby, rond, tafeltennis, pingpong, squash	geduld, mongool, stofwolk, giraffe, kaliber, garde, notitieboekje, afwas, berging, restaurant
blauw	lucht, politie, jeans, politieagent, hemel	beestje, scherf, pastoor, voorschoot, krulhaar, straatmuziek, telefoonkabel, vitrine, slaap, sax
bloem	margriet, vaas, iris, tulp, orchidee	filter, reu, mop, golfstok, strikje, vervuiling, paling, hal, plas, kwal
boom	bos, specht, blad, tak, hout	zoutvat, turntoestel, hitte, conservenblik, krant, huifkar, neerhof, kolonist, schrijfgerei, ruimteschip
boot	anker, schip, hovercraft, varen, vaartuig	handbeweging, arbeid, wandelpad, ijshockey, karwei, vezel, crisis, buste, gereedschap, gasvuur
bos	denneappel, hert, boom, vos, paddestoel	delinquent, rouw, video, doek, praal, slurf, bombardement, horrorfilm, weg, verstand
brief	postzegel, enveloppe, post, brievenbus, postbode	blind, smog, kop, stro, reus, buschauffeur, muntje, hoepel, ingenieur, kunststof
broek	riem, gesp, jeans, rits, kleding	trom, code, oorveeg, spelling, langpootmug, ringetje, jojo, explosief, inlegzool, latex
deur	sleutel, scharnier, klink, deurknop, slot	associatie, springbok, lichaamscel, lappendeken, smaak, tijdmachine, etalagepop, sufferd, opvoeding, platina
dier	poot, staart, gekko, lama, hert	schel, venijn, kenteken, beschermer, zwavel, doelstelling, berging, boef, oneindigheid, kerk
drinken	dorst, glas, bier, vloeistof, water	afloop, beschadiging, walging, tennis, interpretatie, les, grime, vacature, huiskamer, teddybeer
ei	kuiken, kip, spek, struisvogel, omelet	onwetendheid, zonnebloempit, boerenpaard, scheenbeen, borstel, observatie, haar, brie, duvel, bloemenwinkel
eten	keuken, kok, restaurant, microgolf, tafel	postwagen, gevangene, zwaluwnest, chef, vervoeging, postkaart, rugleuning, radio, dragon, speldekop
fiets	tandem, postbode, zadel, vervoer, wiel	stilleven, schmink, pinda, shampoo, stoelgang, illegaal, cruise, gedicht, chocoladetaart, vlaai

Prime	Related probes	Unrelated probes
fruit	sap, appel, peer, perzik, mango	vaccin, sabel, buideldier, loop, koper, laars, grill, singel, smeerkaas, zondag
geel	pudding, parkiet, ananas, kanarie, kaas	blijde, zomervakantie, weemoed, wijze, buffel, tomahawk, chirurgie, levensritme, tooi, sporter
geld	kassa, portefeuille, spaarvarken, portemonnee, kluis	augurk, opener, departement, kar, keyboard, rolschaatsbaan, kunststijl, uitloop, spionage, sereniteit
gitaar	banjo, snaar, rock, straatmuzikant, muziekinstrument	papaver, schrijfmap, ceintuur, kerstboom, schaafwond, kruisteken, brievenbus, keukengerei, verbeelding, scout
gras	tuin, groen, grasmachine, weide, wei	credit, openheid, menseneter, zool, brievenbus, proefje, thuis, salami, golfbeweging, zitkamer
groen	kikker, gras, kiwi, spinazie, komkommer	klimop, vinger, practicum, volwassenheid, lans, hel, punk, impasse, meter, stormram
groente	selderij, aubergine, artisjok, venkel, wittekool	havenwerker, functie, beeld, twist, erfenis, beenhouwer, vergroting, schoolbank, wetgeving, flanel
groot	klein, dinosaurus, reus, olifant, walvis	planner, klank, industrie, prestatie, speelkaart, buidel, badhanddoek, foetsie, meet, onderzoeksinstrument
haar	luis, kuif, hoofd, pruik, kam	niet, klikker, manager, hijs, werkman, nicht, deeg, uier, ontsmettingsmiddel, ara
hout	zaag, plank, splinter, schaaf, boom	tikmachine, delicatesse, loempia, mosterd, grind, tortel, puntschoen, slotenmaker, garagepoort, marktplaats
kaas	muizeval, gat, rasp, geel, sandwich	koffieboon, rendement, lied, zandplaat, geschiedenisles, dokwerker, schoolslag, koude, vereniging, vrieskou
kind	opvoedster, speeltuin, tekenfilm, schommel, draaimolen	bruidstaart, racepaard, schrijfkamer, ruiten, klapband, overjas, aanwezigheid, zeester, rest, hak
klein	mier, microscoop, pony, hamster, groot	schadevergoeding, innerlijk, strijkbout, vuilemmer, roet, gemoedstoestand, petitie, poedel, hijskraan, denim
kleur	paars, kameleon, papegaai, blauw, regenboog	scheikunde, tel, puppy, vlam, flair, elektronica, mijnwerker, gemiauw, loon, toeverlaat
licht	pluimpje, vuurtoren, lamp, schakelaar, zaklamp	wind, gracht, kopstoot, volwassenheid, mergbeen, paleontoloog, aannemer, klaagzang, sjorring, visserij
muis	muizeval, kaas, rat, kat, grijs	slijtage, drietand, wegaanduiding, wijs, funk, ketelmuziek, mogelijkheid, scout, hoorn, biljart
muziek	banjo, notenbalk, synthesizer, ritme, tamboerijn	papa, voorbeeld, emancipatie, zonneschijn, treffen, kleurverandering, strook, hitte, verstopping, natrium
oorlog	soldaat, tank, granaat, vrede, bazooka	smash, avontuur, vislijm, aardbeving, rijder, raad, rappel, plakker, hengst, ven
oranje	pompoen, clementine, sinaasappel, abrikoos, wortel	kristal, spriet, weidsheid, literatuur, prei, adem, evenbeeld, nevel, greep, medaillon

S1.2 Online stimuli

Prime	Related probes	# Probes
wit	wit, zwart, kleur, licht, brood, ei	6
eten	eten, vis, geur, winkel, keuken, pot, oma, vlees, groente, restaurant, schort, tafel, veel, drinken, soep, wild, ei, noot	18
rood	rood, groen, geel, kleur, blauw, bloed, vuur, liefde, vlees, roze, roos	11
zwart	wit, zwart, kleur, dood, grijs, donker, vlieg, hoed, leer, kat	10
water	water, blauw, zee, plant, drinken, nat, plat	7
groot	groot, klein, man, paard, veel	5
groen	rood, groen, kleur, boom, tuin, natuur, gras, plant, oranje, groente, veld	11
zomer	zomer, vakantie, zon, zee, winter, vlieg, fruit, strand	8
pijn	pijn, bloed, vuur, tand, scherp	5
bruin	bruin, boom, hout, haar, paard, grond, kast, noot	8
klein	groot, klein, kind, vlieg	4
werk	werk, saai, geld, schort, beroep, werktuig	6
dier	dier, natuur, bos, mens, hond, paard, vlees, kat, wild	9
kind	klein, kind, man, spel, vrouw, mens, moeder, bescherming, step	9
vakantie	zomer, vakantie, zon, zee, boek, reis, zand, strand	8
geel	rood, geel, kleur, blauw, zon, oranje, licht, bus, kaas	9
kleur	wit, rood, zwart, groen, geel, kleur, blauw, bloem, haar, oranje, roze, roos	12
boom	groen, bruin, boom, hout, tuin, natuur, bos, wind, blad, stok, noot	11
blauw	rood, water, geel, kleur, blauw, zee, lucht, aarde, oog, politie	10
rond	rond, dik, bal, voetbal	4
lawaai	lawaai, geluid	2
vuil	vuil, stof, schort, was	4
hout	bruin, boom, hout, bos, vuur, fluit, stok, kast	8
tuin	groen, boom, tuin, zon, gras, bloem, plant, werktuig	8
natuur	groen, dier, boom, natuur, bos, gras, bloem, plant, berg	9
man	groot, kind, man, vrouw, mens	5
school	school, saai, boek, leer, dom, vriend, bus	7
zon	zomer, vakantie, geel, tuin, zon, zee, reis, zand, licht, strand	10
dood	zwart, dood	2
spel	kind, spel, voetbal, plezier, vriend	5
auto	auto, gevaar, snel, duur, weg, band, snelheid	7
zee	water, zomer, vakantie, blauw, zon, zee, vis, reis, zand, boot, nat, land, strand	13
oud	oud, grijs, oma, hoed	4
bos	dier, boom, hout, natuur, bos, blad, stok, wild, konijn	9
saai	werk, school, saai	3
film	film	1
grijs	zwart, oud, grijs, ijzer, metaal, haar, steen, donker, oma	9
vrouw	kind, man, vrouw, moeder, meisje	5
ijzer	grijs, ijzer, metaal	3
geld	werk, geld, winkel, veel, beroep	5
vis	eten, zee, vis, stank	4
gevaar	auto, gevaar	2
huis	huis, steen	2
geluid	lawaai, geluid	2

Prime	Related probes	# Probes
muziek	muziek, feest, dans, noot	4
gras	groen, tuin, natuur, gras, grond, veld, voetbal	7
lief	lief, oma, vriend, meisje, konijn	5
snel	auto, snel	2
feest	muziek, feest, drank, dans, plezier, vriend	6
mens	dier, kind, man, mens, veel	5
metaal	grijs, ijzer, metaal	3
geur	eten, geur, bloem, stank, roos	5
bloem	kleur, tuin, natuur, geur, bloem, plant, pot, veld, roze, roos	10
duur	auto, duur, restaurant, kleding	4
stank	vis, geur, stank	3
winkel	eten, geld, winkel, zak	4
dik	rond, dik	2
stof	vuil, stof, kleding	3
fiets	fiets, band, step	3
plant	water, groen, tuin, natuur, bloem, plant, pot, blad	8
bloed	rood, pijn, bloed, vlees	4
haar	bruin, kleur, grijs, haar	4
hond	dier, hond, vriend, kat, stok	5
boek	vakantie, school, boek	3
sport	sport, voetbal	2
keuken	eten, keuken, pot, schort, tafel	5
paard	groot, bruin, dier, paard	4
steen	grijs, huis, steen, grond, berg	5
vogel	vogel, lucht, vlieg, ei	4
lucht	blauw, vogel, lucht, wind	4
pot	eten, bloem, plant, keuken, pot	5
winter	zomer, winter, donker, ijs, soep	5
donker	zwart, grijs, winter, donker, licht, gat, middeleeuwen	7
reis	vakantie, zon, zee, reis, weg, strand	6
zand	vakantie, zon, zee, zand, strand	5
oranje	groen, geel, kleur, oranje	4
oma	eten, oud, grijs, lief, oma	5
grond	bruin, gras, steen, grond, gat, aarde, land	7
vlieg	zwart, zomer, klein, vogel, vlieg	5
vuur	rood, pijn, hout, vuur	4
licht	wit, geel, zon, donker, licht	5
gat	donker, grond, gat, kaas	4
berg	natuur, steen, berg, veel	4
hoed	zwart, oud, hoed	3
liefde	rood, liefde, moeder, vriend, roos	5
wind	boom, lucht, wind, regen	4
bal	rond, bal, voetbal, soep	4
drank	feest, drank, glas	3
vlees	eten, rood, dier, bloed, vlees	5
groente	eten, groen, groente, soep, fruit	5
moeder	kind, vrouw, liefde, moeder, schort, bescherming, was	7
veld	groen, gras, bloem, veld, voetbal, boer	6
voetbal	rond, spel, gras, sport, bal, veld, voetbal	7

Prime	Related probes	# Probes
blad	boom, bos, plant, blad	4
tand	pijn, tand, konijn	3
weg	auto, reis, weg	3
boot	zee, boot	2
dans	muziek, feest, dans, plezier	4
plezier	spel, feest, dans, plezier	4
restaurant	eten, duur, restaurant, drinken	4
leer	zwart, school, leer, schoen	4
band	auto, fiets, band, vriend	4
kleding	duur, stof, kleding	3
schort	eten, werk, vuil, keuken, moeder, schort	6
aarde	blauw, grond, aarde	3
dom	school, dom	2
regen	wind, regen, nat	3
roze	rood, kleur, bloem, roze, meisje, roos	6
schoen	leer, schoen	2
brood	wit, brood, kaas	3
ijs	winter, ijs	2
scherp	pijn, scherp, glas	3
tafel	eten, keuken, tafel, kast	4
vriend	school, spel, lief, feest, hond, liefde, band, vriend	8
kat	zwart, dier, hond, kat	4
veel	eten, groot, geld, mens, berg, veel	6
bescherming	kind, moeder, bescherming	3
boer	veld, boer	2
drinken	eten, water, restaurant, drinken, glas	5
meisje	vrouw, lief, roze, meisje	4
nat	water, zee, regen, nat	4
oog	blauw, oog	2
roos	rood, kleur, geur, bloem, liefde, roze, roos	7
step	kind, fiets, step	3
hand	hand	1
plat	water, plat	2
soep	eten, winter, bal, groente, soep	5
beroep	werk, geld, beroep	3
fluit	hout, fluit, noot	3
stok	boom, hout, bos, hond, stok	5
wild	eten, dier, bos, wild	4
bus	geel, school, bus	3
fruit	zomer, groente, fruit	3
glas	drank, scherp, drinken, glas	4
kerk	kerk	1
middeleeuwen	donker, middeleeuwen	2
ei	wit, eten, vogel, ei	4
konijn	bos, lief, tand, konijn	4
land	zee, grond, land	3
zak	winkel, zak	2
kast	bruin, hout, tafel, kast	4
noot	eten, bruin, boom, muziek, fluit, noot	6

Prime	Related probes	# Probes
snelheid	auto, snelheid	2
politie	blauw, politie	2
was	vuil, moeder, was	3
zoet	zoet, suiker	2
kaas	geel, gat, brood, kaas	4
strand	zomer, vakantie, zon, zee, reis, zand, strand	7
suiker	zoet, suiker	2
werktuig	werk, tuin, werktuig	3

S1.3 Post-training stimuli

Prime	Related probes	Unrelated probes
paard	zadel, ruiter, ros, koets, manen	selectie, zeelucht, nicht, inlegzool, index, clochard, namiddag, bekendheid, sluwigheid, handenarbeid
pijn	tandarts, zweep, splinter, verdriet, wond	antwoord, hogeschool, medelijden, moment, mongool, tijdrit, oogschaduw, gezicht, onderverdeling, titanium
regen	nat, regenboog, paraplu, worm, wolk	item, kauw, erwt, gommetje, goliath, speelster, geluid, droefheid, pils, hijs
rijst	wok, paella, kip, wit, eten	kempen, funk, uniform, grind, trainer, tentzeil, bezinning, kruimel, zwijgen, reukorgaan
rood	lippenstift, tomaat, grenadine, klaproos, verkeerslicht	gigant, gymnasium, hasj, laurier, zuid, bloemist, stumperd, sprei, eindpunt, haarborstel
rook	brand, sigaret, tabak, sigaar, schoorsteen	overjas, meetlint, mestkever, citrusvrucht, snijmes, glaswerk, zadeldak, schaaf, bewerking, windwijzer
sneeuw	muts, slede, wit, winter, sneeuwman	huid, wasverzachter, luchtgat, schoorsteenveger, scheldpartij, grasmat, platenspeler, put, object, gerechtszaal
sport	doping, rugby, volleybal, rugbybal, polo	politica, blijdschap, naaldhak, libel, kippenhok, damesschoen, mestkever, trombose, dansvloer, verzekering
stank	ui, stinkdier, vuilbak, vuilniskar, asbak	schok, knaagtand, graat, koffiezetapparaat, atoomreactor, kinderafdeling, tongzoen, stapelplaats, woonwagen, papier
tand	vampier, piranha, tandpasta, krokodil, tandenborstel	schilderij, voeg, folk, gamma, inbreuk, socialist, handtekening, factuur, kruidnagel, slaapkamer
tuin	hek, kruiwagen, gras, hark, bloem	passievrucht, paardestal, zwavelzuur, luider, opzoeking, route, gerommel, lelijkheid, speeltje, bast
vakantie	tent, caravan, krokus, zomer, fototoestel	els, langoustine, uitschot, koe, rijlaars, springpoot, investering, mummie, merg, maandagavond
