# Supplementary material for: Towards a Communication Brain Computer Interface Based on Semantic Relations
Source: PLoS One. 2014 Feb 7;9(2):e87511. doi: 10.1371/journal.pone.0087511 (PMC3917851; doi:10.1371/journal.pone.0087511)
Supplement: File S2 — Results. Additional results. Three figures with additional results. (PDF) [file pone.0087511.s002.pdf]

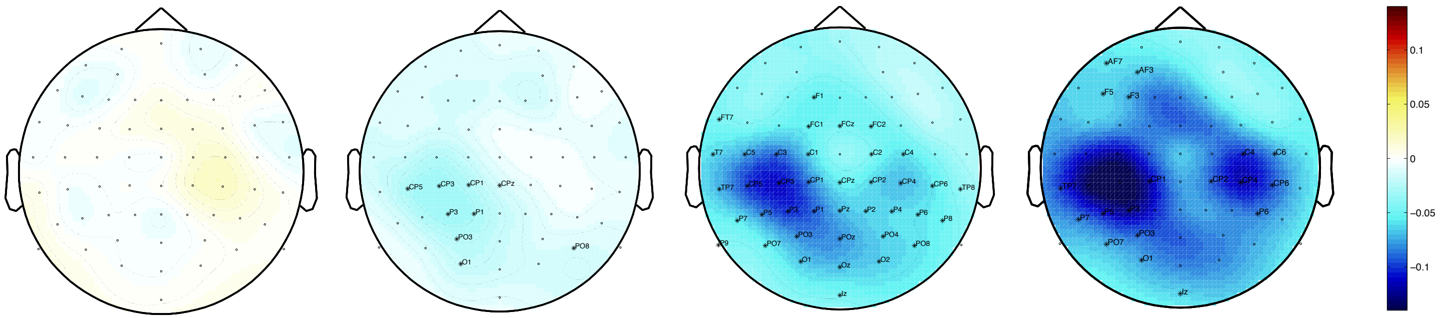

Figure S2.1: **Evolution of band power topography:** Difference topography (related/movement - unrelated/no movement) in the 10–14 Hz frequency band. The respective time windows of the topographies are: 0–250ms, 250–500ms, 500–750ms and 750–1000ms. Asterisks indicate channels with significant differences in all frequency bins between 10 and 14 Hz.

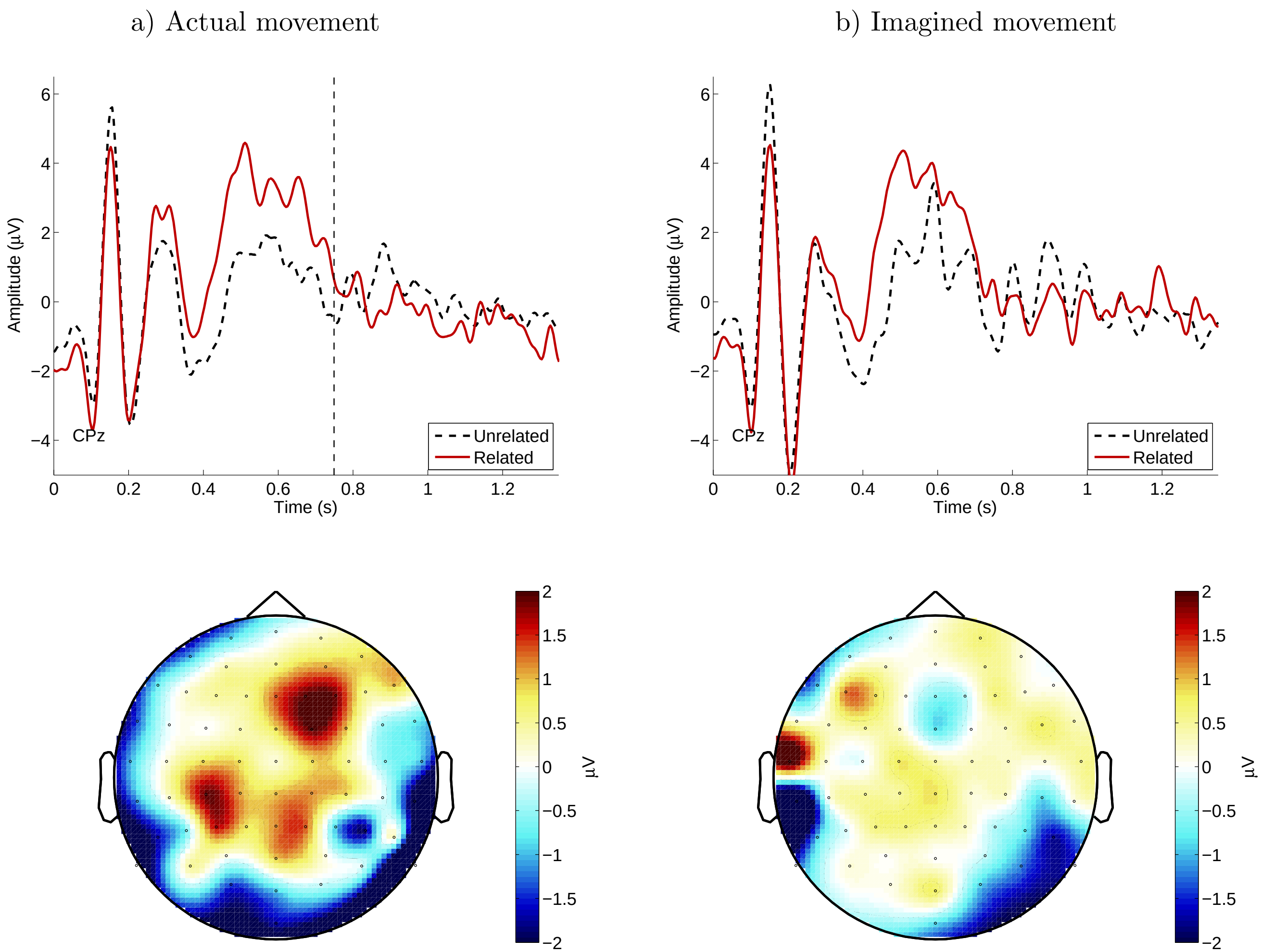

Figure S2.2: **Single subject ERP, actual movement (a) vs imagined movement (b):** Single subject Event Related Potential (ERP) results for the training block. Top: the ERPs for the related (solid red) and unrelated (dashed black) conditions. The dashed vertical line indicates the average reaction time, i.e., when the subjects pressed the button. Bottom: The distribution over the scalp of the significant difference (related – unrelated) averaged over the significant area from the grand average analysis: 260ms – 1000 ms. The topographies show similar structure in both conditions, with a large central positivity (red/white color), surrounded by a peripheral (primarily occipital) negativity.

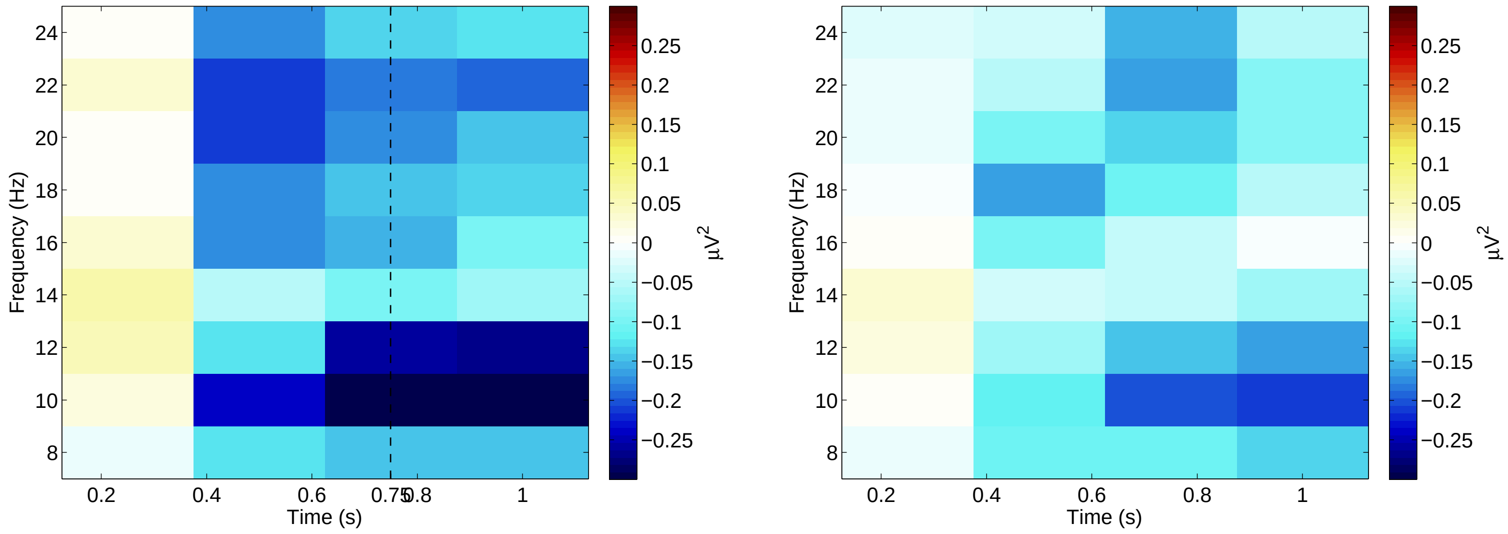

Figure S2.3: **Single subject TFR, actual movement vs imagined movement:** Single subject TFR results for the training block for channel C3. The data shown here is a normalised difference between the related and unrelated conditions, obtained by $\frac{related-unrelated}{related+unrelated}$. The vertical dashed line indicates the grand average reaction time, i.e., when the subjects pressed the button. Left: The actual movement session. Right: the imagined movement session.
